# Supplementary material for: Age-dependent ACE2/TMPRSS2 expression and SARS-CoV-2 household transmission in Gran Canaria
Source: PLoS One. 2025 Jul 29;20(7):e0329229. doi: 10.1371/journal.pone.0329229 (PMC12306786; doi:10.1371/journal.pone.0329229)
Supplement: Attachment — Submitted filename: Response to reviewers. May_25 (2).docx [file pone.0329229.s002.docx]

**Response to Academic Editor and reviewers**

**ACADEMIC EDITOR:**

**Overall Assessment:**
This manuscript presents a population-based, cross-sectional analysis evaluating ACE2 and TMPRSS2 expression in relation to SARS-CoV-2 susceptibility and transmission within households. The study is timely and methodologically sound, with detailed serological and molecular assessments. It adds meaningful insights to the understanding of pediatric susceptibility and receptor biology in COVID-19.

However, a few revisions are required to enhance transparency, clarify methodology, and support key conclusions. Below is a structured editorial assessment:

**Methods and Statistical Analyses.**

- **Concerns:**
  - **Sample size calculation** was not provided, which is critical for assessing power and validity. Please include or justify its absence.

“No formal sample size calculation was performed for this study. The sample size was determined by the number of participants who agreed to participate during the recruitment period. While we aimed to enroll as many participants as possible to maximize statistical power and minimize the risk of type II error, we acknowledge that the absence of a priori sample size estimation may limit the generalizability and statistical robustness of our findings.” **This comment has been added in “Methods” section of the manuscript.**

- Clarify handling of potential **confounding variables**, especially in logistic regression models for transmission risk (e.g., socio-economic status, comorbidities, vaccination status, if relevant).

Socio-economic status was not evaluated in the study. All patients were unvaccinated, given the early stages of the pandemic during which the study was conducted. **This comment has been added in “Methods” section of the manuscript.**

- Discuss the implications of **selection bias** more thoroughly—families with symptomatic individuals may have been more likely to participate.

This paragraph has been added to the Discussion (limitation) section: “it is likely that families with symptomatic individuals or known exposures were more motivated to participate, potentially leading to an overrepresentation of higher-risk households. This selection bias limits the generalizability of our findings to the broader community.”

- **Sample Size Calculations**

**Required Action:** Please include a justification or post hoc power analysis to support the adequacy of the sample size, particularly for subgroup analyses in children.

The following paragraph has been added in the discussion, when describing the limitations of the study: “An important limitation of our study lies in the statistical power of certain subgroup analyses. Although the overall sample size was relatively large, the number of pediatric participants with valid ACE2 and TMPRSS2 expression data was limited. Post hoc power analysis revealed that the comparisons of ACE2 and TMPRSS2 expression between children and adults yielded small effect sizes (Cohen's d = 0.18 and 0.15, respectively), resulting in low statistical power (<25%). These results suggest that the analysis may have been underpowered to detect subtle differences in receptor expression between age groups. In contrast, the comparison of seropositivity rates between children and adults demonstrated a large effect size (Cohen's w = 2.42) and excellent power (>99%). Similarly, the association between age and seropositivity in adults (Cohen's d = 0.42; power = 96%) reinforces the robustness of the age gradient observed in this group. However, the corresponding analysis in children, despite a larger effect size (Cohen's d = 0.57), was only moderately powered (56%), limiting the strength of inference that can be drawn.”

- **Limitations Discussion**

**Suggestions:**

- Discuss the **generalizability** of results, given the unique context of early-pandemic Gran Canaria.

We added this paragraph after the Limitations: “Regarding the generalizability of our findings, it is important to acknowledge that the early-pandemic context and the geographic isolation of the study population may limit extrapolation to other regions or later stages of the pandemic.”

- Clarify that **variant influence** cannot be excluded due to the early sampling period.

We have clarified in the Limitations section that our study was conducted prior to the emergence of major variants such as Delta and Omicron, and that variant-specific effects on transmission and susceptibility cannot be excluded.

- **Additional Recommendations:**
- **Title:** Consider shortening for clarity. Suggested: “Age-dependent ACE2/TMPRSS2 expression and SARS-CoV-2 household transmission in Gran Canaria.” Done
- **Data Availability:** Currently states data available on request. PLOS encourages open data. Please consider uploading de-identified datasets to a public repository if feasible, or strengthen the rationale for restrictions.

The following paragraph has been added to the “availability of data and material statement”: The data contain potentially identifying information from a small and geographically confined population, and public sharing was not included in the informed consent approved by the ethics committee. Therefore, the full dataset cannot be made publicly available. However, de-identified data may be made available to qualified researchers upon reasonable request, subject to approval by the ethics committee of the Complejo Hospitalario Universitario Insular-Materno Infantil and compliance with applicable data protection regulations.

==============================

Please submit your revised manuscript by Jun 17 2025 11:59PM. If you will need more time than this to complete your revisions, please reply to this message or contact the journal office at [plosone@plos.org](mailto:plosone@plos.org). When you're ready to submit your revision, log on to https://www.editorialmanager.com/pone/ and select the 'Submissions Needing Revision' folder to locate your manuscript file.

Please include the following items when submitting your revised manuscript:

- A rebuttal letter that responds to each point raised by the academic editor and reviewer(s). You should upload this letter as a separate file labeled 'Response to Reviewers'. done
- A marked-up copy of your manuscript that highlights changes made to the original version. You should upload this as a separate file labeled 'Revised Manuscript with Track Changes'. done
- An unmarked version of your revised paper without tracked changes. You should upload this as a separate file labeled 'Manuscript'. done

If you would like to make changes to your financial disclosure, please include your updated statement in your cover letter. Guidelines for resubmitting your figure files are available below the reviewer comments at the end of this letter.

Journal Requirements:

When submitting your revision, we need you to address these additional requirements.

1. Please ensure that your manuscript meets PLOS ONE's style requirements, including those for file naming. The PLOS ONE style templates can be found at https://journals.plos.org/plosone/s/file?id=wjVg/PLOSOne_formatting_sample_main_body.pdf and <https://journals.plos.org/plosone/s/file?id=ba62/PLOSOne_formatting_sample_title_authors_affiliations.pdf> Done

  2. We note that the grant information you provided in the ‘Funding Information’ and ‘Financial Disclosure’ sections do not match. When you resubmit, please ensure that you provide the correct grant numbers for the awards you received for your study in the ‘Funding Information’ section. Done

 3. Your ethics statement should only appear in the Methods section of your manuscript. If your ethics statement is written in any section besides the Methods, please move it to the Methods section and delete it from any other section. Please ensure that your ethics statement is included in your manuscript, as the ethics statement entered into the online submission form will not be published alongside your manuscript. DONE

 4. We note that you have indicated that there are restrictions to data sharing for this study. For studies involving human research participant data or other sensitive data, we encourage authors to share de-identified or anonymized data. However, when data cannot be publicly shared for ethical reasons, we allow authors to make their data sets available upon request. For information on unacceptable data access restrictions, please see <http://journals.plos.org/plosone/s/data-availability#loc-unacceptable-data-access-restrictions>. Addressed in the Declarations section of the manuscript

Before we proceed with your manuscript, please address the following prompts:

a) If there are ethical or legal restrictions on sharing a de-identified data set, please explain them in detail (e.g., data contain potentially identifying or sensitive patient information, data are owned by a third-party organization, etc.) and who has imposed them (e.g., a Research Ethics Committee or Institutional Review Board, etc.). Please also provide contact information for a data access committee, ethics committee, or other institutional body to which data requests may be sent. DONE

Please update your Data Availability statement in the submission form accordingly.

  5. In the online submission form, you indicated that the datasets generated and/or analyzed during the study are not publicly available due to participant confidentiality, but are available from the corresponding author upon reasonable request. All PLOS journals now require all data underlying the findings described in their manuscript to be freely available to other researchers, either 1. In a public repository, 2. Within the manuscript itself, or 3. Uploaded as supplementary information. This policy applies to all data except where public deposition would breach compliance with the protocol approved by your research ethics board. If your data cannot be made publicly available for ethical or legal reasons (e.g., public availability would compromise patient privacy), please explain your reasons on resubmission and your exemption request will be escalated for approval. DONE

 Reviewers' comments:

**Reviewer #1:** The authors aimed to assess whether the expression of ACE2 and TMPRSS2 is associated with susceptibility to and severity of COVID-19 across age groups. The study is well designed but vaccination status of the participant did not mentioned. So authors should mention it. DONE

Also which variant was observed duration of the study in the region? The ancestral Wuhan strain. It has been added both in the methodology and discussion sections

ACE2 is located on X chromosome so males are hemizygous for the ACE2 gene. Males and females might have different levels of ACE gene expression. This information and expression results for both gender should be discussed. We thank the reviewer for this important observation. As correctly noted, ACE2 is located on the X chromosome and sex-linked differences in expression are biologically plausible. In our analysis, we included sex as a variable when examining ACE2 and TMPRSS2 expression. However, no statistically significant differences were found between males and females for either gene (ACE2: median 114 vs. 109; p = 0.948; TMPRSS2: median 100 vs. 90; p = 0.128). These results are detailed in Table 6. While our findings do not support sex-related differential expression in saliva samples, we acknowledge that other tissues or regulatory mechanisms (e.g., X inactivation escape, hormonal regulation) may yield different results and warrant further study. We have clarified this point in the discussion section.

Why the authors did not check the ACE2 and TMPRSS2 gene variants that also effect the receptor structure? If they can also discuss it it will give more scientific value to the manuscript.

We thank the reviewer for raising this important point. Indeed, ACE2 and TMPRSS2 genetic variants may influence receptor structure and viral entry, and several polymorphisms have been associated with differential susceptibility to SARS-CoV-2 infection. However, our study was designed to evaluate gene expression levels in saliva and did not include sequencing or genotyping components. We had briefly acknowledged this in the Discussion and have now expanded that section to clarify that genetic variability in these genes could contribute to host susceptibility independently of expression levels and merits further investigation.

**Reviewer #2:** Age-related ACE2 and TMPRSS2 expression and household transmission of SARSCoV-2:Insights from a population-based study in Gran Canaria
After reviewing the research and its findings, I find that the study is well-conducted and its results are accurate and valid. Therefore, I recommend its acceptance in its current form. We thank the reviewer for their positive assessment.

**Reviewer #3:** Poch-Pàez and colleagues describe a cross-sectional study conducted in Gran Canaria between March 10, 2020 and June 2, 2020 evaluating age-related ACE2 and TMPRSS2 gene expression (saliva samples) and household transmission of SARS-CoV2. Their data encompass 258 households with 650 individuals (of whom 89 were children <18 years of age). Their findings did not support a strong relationship between expression of these 2 genes and susceptibility or severity of COVID-19 and noted that children appeared to be less susceptible to SARS-COV-2 infection and to have milder disease.

The study is interesting, although somewhat confusing in places. Overall, it’s value currently is diminished by the many other published studies on transmission dynamics, the emergence of many newer variants with differing infectivities and severities of infection, the introduction of COVID-19 vaccinations, and the shrinking pool of SARS-CoV-2 naïve adults and children.

I have some questions and concerns that the authors should address:
1) The study was conducted early in the pandemic (March to June 2020). Their statement in the Background section that “published data point toward similar (7) or even lower (8,9) infection compared to adults” when referring to pediatric SARS-CoV-2 infection uses references published in 2020 (again, early in the pandemic) and does not consider how infectivity of other SARS-CoV-2 variants differ from the initial clades. For example, in a CDC study that compared seroprevalence rates between September-December 2021 to January-February 2022 (emergence of Omicron B.1.1.529 variant), seroprevalence among children 0-11 years of age increased from 44.2% to 75.2%, and from 45.6% to 74.2% among those aged 12-17 years, respectively. Among adults ages 18-49 years, seroprevalence increased from 36.5% to 63.7%, and from 28.8% to 49.8% among those 50-64 years of age. The CDC study showed that children had higher SARS-CoV-2 seroprevalence than adults. This difference from the current authors’ results is likely due to the infectivity of circulating SARS-CoV-2 variants at the time of the study, and probably geographic differences. [Clark et al. MMWR Morb Mortal Wkly Rep 2022;71:606-8]. Thank you for your suggestion. We tried to reflect this by citing Khemiri et als work (Khemiri H, Ayouni K, Triki H, Haddad-Boubaker S. SARS-CoV-2 infection in pediatric population before and during the Delta (B.1.617.2) and Omicron (B.1.1.529) variants era. Virol J. 2022;19(1):144.) but have expanded added the new suggested reference.

2) In the Methods-Data and Sample Collection, did the authors collect data on socioeconomic status of the 258 household “clusters”? Higher socioeconomic status is generally associated with lower SARS-CoV-2 antibody prevalence [Naeimi et al. eClinical Medicine 2023;56:101786].

We did not. This has also been suggested by other reviewers. We clarified this point in the methodology section.

3) Similarly, do we know how many individuals were diabetic, since the condition is associated with higher ACE2 expression in many tissues (lungs, kidneys, pancreas, etc)? Conversely, patients with allergic sensitization and asthma have lower ACE2 gene expression [Shukla. Eur Arch Otorhinolaryngol 2020;278:2637-40].

We thank the reviewer for highlighting the potential impact of specific comorbidities such as diabetes and allergic conditions on ACE2 expression. We did collect data on the presence of chronic comorbidities, including diabetes and asthma. However, due to the limited number of individuals with each specific condition, we grouped comorbidities into a single composite variable to avoid underpowered subgroup analyses. In our multivariate logistic regression, the presence of comorbidities was not significantly associated with ACE2 or TMPRSS2 expression. We acknowledge that this approach may have masked condition-specific effects, particularly for conditions such as diabetes and asthma. These relationships merit further investigation in studies with larger and more stratified cohorts. We have added this point to the discussion, with the inclusion of the suggested reference.

4) Lactobacilli colonizing the respiratory tract can down-regulate ACE2 expression. Rates of colonization in infants, children, and adults vary. This was not examined as a possible variable. [Taufer and Rampeletto. Microorganisms 2024;12:284]

We thank the reviewer for highlighting this interesting and emerging aspect. However, our study did not include microbiome profiling or bacterial culture data and was not designed to evaluate microbial influences on gene expression. We agree that this represents a promising area for future research, and have added a brief comment in the Discussion to reflect this point.

5) Is “sharing a bedroom” the same as sharing a bed (Transmission Dynamics section)? No. we meant sharing a bedroom. It has been modified in the Methods section for clarity. Thank you for your suggestion.

6) In Table 5, it would be helpful to show the amount of ACE2 and TMPRSS2 gene expression between those who are IgG positive and those who are not. The information has been added.

7) In the title of Table 5, please change “ECA2” to “ACE2”. Also, the same error appears in Table 6, row 3. Thank you for catching that. It has been modified.

8) Were schools/day cares closed in Gran Canaria during the study period, as this could provide another explanation for the lower seropositivity in children. What kind of infection control/isolation advice was given at the time to infected index cases (e.g., masking, self-quarantine, staying alone in a room)? Was there any difference in household transmission rates among those who were hospitalized/died (less exposure time to family members) versus those who were not?

We thank the reviewer for this comment. Regarding school attendance, during the initial phase of the pandemic in Gran Canaria—corresponding to our study period—children continued attending school or daycare as usual. Widespread closures had not yet been implemented, which may have contributed to early exposures. However, most pediatric index cases were identified later in the course of household outbreaks, after adult cases had been detected.

With respect to infection control advice, index cases were counseled—when possible—to self-isolate in a separate bedroom and bathroom, and to minimize contact with household members. However, it is important to note that many index cases in our cohort were among the first individuals diagnosed in their households and in the broader community, at a time when public health guidance was still evolving. As a proxy for close contact and potential failure to isolate, we recorded whether household members shared a bedroom with the index case

We did analyze hospitalization status and comorbidities as a potential factor influencing transmission. However, hospitalization of the index case was not associated with a statistically significant difference in household transmission rates in our multivariate model.

We added this paragraph in the Methods section to address these concerns: “During the study period (March–June 2020), formal infection control guidance was still evolving in Gran Canaria. Index cases were advised to isolate in a separate bedroom and bathroom when feasible, but adherence to these recommendations was not systematically assessed. As a proxy for close contact and potential failure to isolate, we recorded whether household members shared a bedroom with the index case. School and daycare closures had not yet been implemented at the time of data collection, and children continued attending educational institutions under normal conditions.”

9) Studies have revealed genetic polymorphisms of ACE1, ACE2, IFTM3, TMPRSS2 and TNFα genes associated with increased risk of infection and illness severity. We do not know what polymorphism are present in the individuals described in this study, both infected and uninfected. [Möhlendick et al. Pharmacogenet Genomics 2021;31:165-71; Pecoraro et al. Clin Exp Med 2023;23:3251-64]

Thank you for your suggestion. Indeed, ACE2 and TMPRSS2 genetic variants may influence receptor structure and viral entry, and several polymorphisms have been associated with differential susceptibility to SARS-CoV-2 infection. However, our study was designed to evaluate gene expression levels in saliva and did not include sequencing or genotyping components. We have briefly acknowledged this in the Discussion and have now expanded that section to clarify that genetic variability in these genes could contribute to host susceptibility independently of expression levels and merits further investigation. We have also included the suggested references.

**Reviewer #4:**

1. Saliva‑based RT‑PCR quantification of ACE2/TMPRSS2 may not reflect receptor abundance in the nasopharynx or lower airway epithelium, where viral entry occurs. Include a paragraph comparing saliva to nasal/nasopharyngeal sampling, citing relevant literature.

We thank the reviewer for this important and insightful comment. We have revised both the Introduction and Discussion to better address tissue-specific expression of ACE2 and TMPRSS2 and the limitations of saliva-based sampling. In the Introduction, we have added the following sentence to clarify the tissue-specific expression pattern of ACE2/TMPRSS2 and its potential age-related implications: “their expression varies by anatomical site, with the highest levels observed in the nasal epithelium, followed by the oral cavity, and lower expression in the lower airway”, including relevant literature.

In the Discussion, we have expanded our analysis to include a new paragraph comparing saliva to other sampling methods, supported by recent literature: “These discrepancies may reflect tissue-specific variability, including differential expression of ACE2 and TMPRSS2 in the nasopharynx, oral mucosa or lower airway epithelium, or as well as technical differences in sample collection. Saliva-based assessments may not fully represent receptor expression in tissues more directly involved in viral entry (5), and results should therefore be interpreted with caution”.

2. "ECA2” appears in Table 6 header instead of “ACE2". Thank you, it has been corrected.
3. “TMPRSS22” instead of “TMPRSS2” in Keywords and several figure legends. Thank you, it has been corrected.
4. Reference formatting: check consistency of journal names (some are abbreviated, some full). Thank you for your comment. We have revised all references to ensure that journal names are consistently formatted according to the National Library of Medicine (NLM) standard abbreviations, as required by PLOS ONE and Vancouver style. All journal names now use the NLM-approved form. Please, bear in mind that for single-word journal titles like *Science*, *Cell*, *Lancet*, *JAMA*, *Viruses*, etc… the NLM abbreviation is the same as the full journal title
5. Numerous hypothesis tests (Tables 3–6, correlations, subgroup comparisons) are reported at p<0.05 without correction. Justify why unadjusted p‑values are acceptable. Report adjusted p‑values for the most important findings (e.g., ACE2 vs. age correlations).

Thank you for your insightful comment. We have now applied the Bonferroni correction to the correlation analyses between ACE2 expression and age in the total population, as well as in the children and adult subgroups (three tests in total). After correction, the previously significant associations no longer meet the conventional threshold for statistical significance (p= 0.06 for the total population, p= 0.105 for children, p= 1.00 for adults). We have updated the Results and Discussion sections to transparently report both unadjusted and adjusted p-values, and to discuss the implications of these findings. We now interpret these associations as exploratory and hypothesis-generating, and we have revised our conclusions accordingly.

**Reviewer #5:** Summary:
This paper examines gene expression as it relates to SARS-CoV-2 household transmission. A cross-sectional study among households was conducted to evaluate gene expression and symptoms among index and household contacts. Although the methodology seems solid, there needs to be more contextualization of the work in this setting and a better sense of the population, target population, and sociodemographic context. The authors could also expand on the implications of this study in terms of how it relates to other island countries and similar populations. The discussion could be condensed to focus more on the main points and how they relate to the literature, with clear sections involving key findings and how they relate to the literature, limitations, and strengths of the study. Also, since this study was done before the Delta and Omicron waves, that should be noted clearly in the limitations section. Also, all of the graphs below should be optimized for individuals with colorblindness, and have clear legends with titles and labeled axes. Finally, this manuscript would benefit from a great deal of copy editing and proofreading to help improve grammatical issues

Specific comments
Page 5 – remove the comma between Some and studies. Done
Page 5 – move the (8,9) citation to after the period after adults. Done
Page 6 – it may be helpful to add some information about the size of Gran Canaria, and the population of children and adults. This information has been added: “Gran Canaria is the third largest of the Canary Islands, covering approximately 1,560 km², with a population of about 875,000 as of 2025. Children under 18 years make up an estimated 15–18% of the population (roughly 130,000–160,000), with the remainder being adults”.
Page 11 – be sure to proofread the table, and make sure that parentheses match for the group age row. Thank you, we have carefully reviewed the table and confirmed that all parentheses are correctly matched and formatted. If there is a specific instance or value you believe may be in error, please let us know and we will address it promptly
Page 12 – you should be specific for your serologic results and indicate if these were among household contacts or the index cases. For example, you could say that, Among household contacts, seroprevalence was 42%. Thank you for your observation. We have modified the current version to reflect your suggestion.

In table 3 on Page 13, you should include the 95% Confidence interval for each odds ratio. If you add that, you will not have to add the p value, and then you can bold or add a * to all of the odds ratios that are statistically significant.
For the symptoms that presented significantly more in adults than in children, you should provide these results in a separate table or in the appendix.

Thank you for your helpful suggestions regarding Table 3. We have updated the table to include 95% confidence intervals for each odds ratio and have indicated statistically significant differences by adding an asterisk (*) both after the symptom name and next to the CI. For symptoms where the odds ratio could not be estimated due to zero cases in one group, we have marked these as “not estimable” (NE) in the table with an explanatory footnote.

We believe that presenting all symptoms in a single table allows for clearer and more direct comparison between adults and children, and helps readers to easily visualize the overall symptom profile. Splitting the data into separate tables or appendices might reduce clarity and make interpretation more cumbersome. We hope this format addresses your concerns while maintaining the clarity and accessibility of the result.

Page 17, all of this information about the sampling and its limitations should be moved to a separate limitations section.

Thank you for your suggestion. We have revised the manuscript to address this point. The discussion section now focuses on interpreting our findings and comparing them with those of Pollán et al., while the limitations related to study design, sampling, and contextual factors have been moved and expanded in a dedicated limitations section.

In general, the discussion section is very long and could be streamlined. I would suggest picking three main findings to focus on, and then asses how those findings compare to the literature.

Thank you. We have followed your suggestions.

Page 21 – You should add information about how these results cannot be extrapolated to the population as a whole because of the study, and may reflect a sicker population than the population of Gran Canaria as a whole. Thank you for your suggestion. We have added the following sentence to the discussion: “our results should not be extrapolated to the general population of Gran Canaria, as our study design and recruitment methods may have selected for households with higher rates of illness or concern about COVID-19, potentially reflecting a sicker or more exposed subgroup than the general population”.

Also, is there any possibility of waning genetic expression over time given that these tests may have been conducted up to 4 months after initial infection? Recent evidence shows that while ACE2 and TMPRSS2 gene expression is temporarily reduced during the acute phase of SARS-CoV-2 infection, expression levels return to baseline after recovery and do not remain suppressed months after infection (***Hui et al. ACE2 and TMPRSS2 gene expression is reduced acutely in SARS-CoV-2 patients but returns to normal with recovery. Sci Rep 2025 Apr 14;15(1):12828. doi: 10.1038/s41598-025-96279-x.*** ). Therefore, it is unlikely that the timing of our tests (up to four months post-infection) would be significantly affected by persistent changes in the expression of these entry factors.

Page 21 – conclusion, you should reiterate that your study found these things, and that more research is needed.

Thank you for your valuable suggestion. We have rephrased the conclusions to more clearly emphasize our key findings and the need for further research, improving clarity and impact.

Pages 38-42 – all of these graphs should have legends and be optimized for black and white printing or red/green colorblindness. Thank you for your suggestion. We have modified the color scheme of the graphs.
